# An Argument for Reconsidering the Role of Social Support in Treating Anxiety Disorders

**DOI:** 10.20900/jpbs.20210010

**Published:** 2021-06-28

**Authors:** Erica A. Hornstein, Naomi I. Eisenberger

**Affiliations:** Department of Psychology, University of California, Los Angeles, 1285 Franz Hall, Los Angeles, CA 90095, USA

**Keywords:** social support, safety signal, fear extinction, exposure therapy, anxiety disorder

## Abstract

Reminders of loved ones have long been avoided during extinction-based treatments because of their assumed status as safety signals, which, by inhibiting fear in the moment, impair the long-term outcomes of fear extinction. Yet, recent work has demonstrated that in contrast to standard safety signals, social support reminders actually enhance fear extinction and lead to lasting reduction of fear, suggesting that they may have beneficial effects during exposure therapy that have before-now been overlooked. Here, we argue for a revision of the assumption that social support is detrimental to fear extinction processes and propose that future work should focus on the potential of social support reminders to improve treatment outcomes in those with anxiety disorders.

Across the world, the number of individuals who suffer from an anxiety disorder has been steadily increasing, with one in three adults in the US expected to receive diagnoses such as Generalized Anxiety Disorder, Social Anxiety Disorder, or Phobia during the course of their lifetime [1,2]. Although significant advances have been made in methods to treat these disorders, even the most successful treatments to-date, exposure therapies (a series of procedures based on fear extinction processes: [3,4]), are limited. While more successful than other treatments, exposure procedures are extremely aversive to complete, leading to high levels of drop-out or low engagement with therapeutic procedures [3–5], and are not always effective, leaving relapse all too common [3,4]. However, recent, surprising findings suggest that reminders of loved ones (pictures of social support figures) may actually serve to enhance fear extinction and alleviate fear symptoms [6–8]—reducing the aversiveness of exposure procedures, easing drop-out, and enhancing the fear extinction processes upon which exposure procedures are based, mitigating relapse. These findings are surprising because they challenge current views of social support reminders as belonging to the category of safety signals, which are known to prevent fear extinction [9,10] and impair exposure therapy treatments [10,11]. However, it appears that while social support reminders and safety signals are similar in that they can *inhibit fear while present*, social support reminders have the additional ability of *reducing fear in the long-term*. Thus, here, we propose that this unique combination of effects calls for a revision of the assumption that social support is detrimental to fear extinction processes and suggests that the role of social support in the treatment of anxiety and dysfunctional fear may have been overlooked.

## EVIDENCE FOR THE BENEFICIAL ROLE OF SOCIAL SUPPORT REMINDERS DURING FEAR EXTINCTION

**Figure 1.**
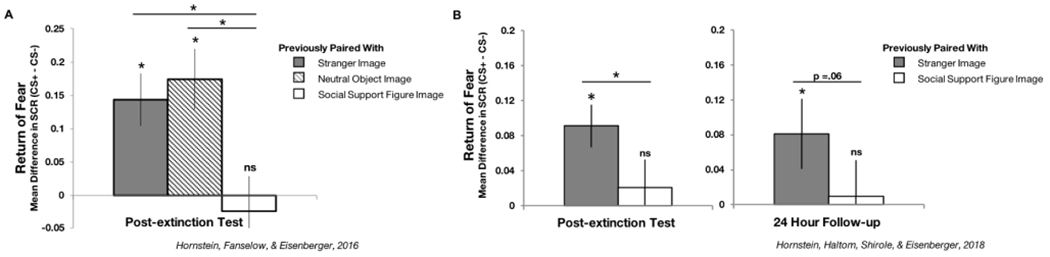
Results from two studies showing the beneficial effects of social support on fear extinction outcomes. (**A**) In a study in which fear extinction was conducted in the presence of images of social support figures (selected by participants and rated as highly supportive), images of neutral objects, and images of strangers (gender, age, and ethnicity matched to the social support figures), results revealed that directly post-extinction, when fear cues were presented on their own once more, return of fear occurred for fear cues previously paired with images of strangers or neutral objects, but not for fear cues previously paired with images of social support figures (see: ref. [6]). (**B**) Similarly, in a study in which fear extinction was conducted in the presence of images of social support figures or strangers, results revealed that directly post-extinction, when fear cues were presented on their own once more, return of fear occurred for fear cues previously paired with images of strangers, but not for those previously paired with images of social support figures. Notably, this pattern of effects persisted even 24-hours-later and following a test designed to reinstate conditioned fears (fear reinstatement: [17]). Specifically, return of fear occurred for fear cues that had been paired with images of strangers the day prior, but not for those that had been paired with images of social support figures (see: ref. [8]).

When a cue that is associated with an aversive outcome (fear cue) is repeatedly presented in the absence of that aversive outcome, new learning occurs that weakens the fear association, and thus the fear response triggered by the cue [12]. This process is known as fear extinction and is one of the most effective ways to reduce both healthy and dysfunctional fears, serving as the basis for exposure therapies [3,11,13]. When other cues are present during this process, their added properties can influence how and whether learning occurs. In the case of safety signals (varying cues that have been learned to signal the absence of a certain aversive outcome and therefore inhibit the fear response brought about by expectation of that outcome: [14]), because they reduce expectation that an aversive outcome will occur, their presence prevents the learning processes that weaken fear associations [15], preventing fear extinction from occurring [9,10]. Social support reminders, because they inhibit the fear response (during fear learning: [6]; during social buffering: [16]), meet the parameters of belonging to the safety signal category. Yet, surprisingly, recent work has demonstrated that while social support reminders do in fact inhibit fear responding [6] like safety signals, they have the additional effect of enhancing fear extinction [6–8], in contrast to safety signals. Indeed, while safety signals prevent any long-term reduction in fear responding, social support reminders lead to fear reduction that lasts longer than if fear extinction is conducted in the presence of images of neutral objects [6], images of strangers [6,7], or pretrained safety signals [8], with no return of fear following extinction procedures or even 24-hours-later following a test designed to reinstate fear (please see Figure 1). This distinct and never-before-seen combination of effects suggests that social support reminders are not engaging the same mechanisms as safety signals during fear learning processes and thus should not be assumed to belong in the safety category. Therefore, social support reminders warrant closer examination, especially with respect to potential benefits during treatments to reduce dysfunctional fear.

## POTENTIAL MECHANISM FOR THE DISTINCT EFFECTS OF SOCIAL SUPPORT

Before considering their implications, it is important to consider how social support reminders bring about their distinct combination of fear-reducing effects. The answer to this may lie in the crucial role of social support figures for survival. Specifically, due to the care, resources, and security provided by close others, social support figures may have evolved to play a unique role in the fear learning circuit, enabling social support to influence fear learning outcomes [6–8,18] and also protecting social support figures from becoming associated with fear [6,19,20]. Of particular note is the fact that the neurobiological processes that have evolved to promote social bonds appear to intersect with the processes that support fear learning, with the opioid system playing a central role in both reinforcing and maintaining social bonds (social-connection-triggered-opioid-release provides a sense of reward and buffers against pain and stress: [21,22]) and driving the calculations that support fear acquisition and extinction (fear-triggered-opioid-release provides the negative feedback necessary for error-correction, leading to the weakening of fear associations during fear extinction: [15,23]). This overlap may provide a point of intersection by which social support is able to directly alter activity in the fear learning circuit by triggering opioid release during fear learning, mimicking fear-triggered-opioid-release and consequently increasing negative feedback and enhancing fear extinction. This explanation would also explain the contrasting effects of social support reminders and safety signals on fear extinction, as safety signals have the contrasting effect on the opioid system, decreasing opioid sensitivity [24] as opposed to triggering opioid release like social support reminders do. Importantly, the opioid system is also known to modulate threat-responding and anxious behaviors [25–27] even outside of the fear learning context, suggesting that, in acting on this system, social support may be particularly beneficial for individuals suffering from dysfunctional fear and anxiety.

## IMPLICATIONS FOR THE ROLE OF SOCIAL SUPPORT FIGURES IN TREATING ANXIETY DISORDERS

The emerging evidence that social support is uniquely able to simultaneously inhibit fear and enhance fear extinction has important implications. As noted above, exposure therapies implement fear extinction procedures to reduce the dysfunctional fears that are hallmark of anxiety disorders and represent the most successful treatments for anxiety to date [3,4]. Yet, these treatments are limited by how aversive they are to complete and the occurrence of relapse [3–5]. While safety signals might be able ease the discomfort of therapeutic procedures, inhibiting fear in the moment, they harm long-term treatment outcomes and have therefore been excluded from treatment settings [3,11,13]. Because social support figures have been placed in the category of safety signals and assumed to have the same effects, social support reminders have likewise been excluded from treatment settings. However, the ability of social support reminders to inhibit fear while also enhancing fear extinction [6–8] not only distinguish them from safety signals, but also indicate that social support may benefit, and not harm, individuals undergoing exposure therapy. Indeed, social support reminders may provide a unique advantage during exposure procedures by enhancing extinction, augmenting therapeutic outcomes, while also reducing the aversiveness that makes such procedures difficult to complete. If this is the case, it is possible that something as simple as an image of a close other during the exposure portion of therapeutic procedures may not only boost treatment compliance and completion, but also lead to longer lasting reductions in anxiety symptoms. Work is needed to examine these possibilities, but the first step of un-yoking social support from the safety category will erase previous assumptions and open the door to new avenues of research. This revision of views on the role of social support during fear learning and anxiety treatment is not only required based on scientific evidence, but is called for in the search for methods to improve treatments for those suffering from anxiety disorders.
